# A Role for Adipocytes and Adipose Stem Cells in the Breast Tumor Microenvironment and Regenerative Medicine

**DOI:** 10.3389/fphys.2021.751239

**Published:** 2021-11-29

**Authors:** Courtney K. Brock, Katherine L. Hebert, Maria Artiles, Maryl K. Wright, Thomas Cheng, Gabrielle O. Windsor, Khoa Nguyen, Madlin S. Alzoubi, Bridgette M. Collins-Burow, Elizabeth C. Martin, Frank H. Lau, Bruce A. Bunnell, Matthew E. Burow

**Affiliations:** ^1^Section of Hematology and Oncology, Department of Medicine, Tulane University School of Medicine, New Orleans, LA, United States; ^2^Department of Microbiology, Immunology, and Genetics, University of North Texas Health Science Center, Fort Worth, TX, United States; ^3^Department of Biological and Agricultural Engineering, Louisiana State University, Baton Rouge, LA, United States; ^4^Section of Plastic & Reconstructive Surgery, Department of Surgery, Louisiana State University Health Sciences Center, New Orleans, LA, United States

**Keywords:** breast cancer, tumor microenvironment, adipocyte, adipose stem cell, obesity, regenerative medicine

## Abstract

Obesity rates are climbing, representing a confounding and contributing factor to many disease states, including cancer. With respect to breast cancer, obesity plays a prominent role in the etiology of this disease, with certain subtypes such as triple-negative breast cancer having a strong correlation between obesity and poor outcomes. Therefore, it is critical to examine the obesity-related alterations to the normal stroma and the tumor microenvironment (TME). Adipocytes and adipose stem cells (ASCs) are major components of breast tissue stroma that have essential functions in both physiological and pathological states, including energy storage and metabolic homeostasis, physical support of breast epithelial cells, and directing inflammatory and wound healing responses through secreted factors. However, these processes can become dysregulated in both metabolic disorders, such as obesity and also in the context of breast cancer. Given the well-established obesity-neoplasia axis, it is critical to understand how interactions between different cell types in the tumor microenvironment, including adipocytes and ASCs, govern carcinogenesis, tumorigenesis, and ultimately metastasis. ASCs and adipocytes have multifactorial roles in cancer progression; however, due to the plastic nature of these cells, they also have a role in regenerative medicine, making them promising tools for tissue engineering. At the physiological level, the interactions between obesity and breast cancer have been examined; here, we will delineate the mechanisms that regulate ASCs and adipocytes in these different contexts through interactions between cancer cells, immune cells, and other cell types present in the tumor microenvironment. We will define the current state of understanding of how adipocytes and ASCs contribute to tumor progression through their role in the tumor microenvironment and how this is altered in the context of obesity. We will also introduce recent developments in utilizing adipocytes and ASCs in novel approaches to breast reconstruction and regenerative medicine.

## Introduction

Between 1999 and 2018, the prevalence of obesity in the United States climbed from 30.5 to 42.3% (Hales et al., 2020). Obesity represents an increasingly common confounding factor to many diseases and conditions, including cancer. Patients with a high body mass index (BMI) have an increased risk of developing many adult cancers (Renehan et al., 2008). Given the prevalence of obesity, it is critical to examine the cellular components of obesity that contribute to these epidemiological trends. Adipocytes, the major cellular component of adipose tissue, and adipose-derived mesenchymal stem cells (ASCs) may be key players. For breast cancer specifically, obesity at diagnosis is associated with higher risk of total mortality and morbidity, as well as worse disease free-survival and overall survival (Lohmann and Goodwin, 2021). Furthermore, increased BMI is associated with more aggressive tumor types and a higher risk of recurrence for breast cancer patients (Zhao et al., 2020). In breast cancer, the proximity of tumors to adipose tissue highlights the need to determine how adipose contributes to the tumor microenvironment.

ASCs and adipocytes are normal components of breast tissue anatomy that provide support through providing energy, hormonal regulation, and cytokines involved in wound healing. In obesity, these cells become altered. Patients with higher BMI have an increased amount of dysfunctional adipocytes leading to gene alteration in the expression profile, inducing inflammation and hypoxia as well as apoptosis. Select patient characteristics have been implicated in variations in ASC expression and ASC contributions to cancer development. Obesity is associated with excessive free fatty acids, cholesterol, triglycerides, hormones such as leptin, interleukins, and chemokines, which all have roles in breast cancer development (Chu et al., 2019).

Interestingly, these cell types may act as a double-edged sword: There is evidence that ASCs, through their interactions with breast cancer cells and immune cells, contribute to tumor progression; however, there have also been promising developments in the use of ASCs in breast reconstruction and tissue engineering. Therefore, it is critical to understand the cell-cell interactions in the tumor microenvironment that contribute to these dual functions. In this review, we will characterize both adipocytes and adipose stem cells’ role in normal physiology and the breast cancer tumor microenvironment, particularly in the context of obesity. Exploring the many roles of ASCs allows for the creation of laboratory models that better address the complexity of the breast cancer tumor microenvironment. Therefore, we will also discuss recent developments in using ASCs in a novel approach to breast reconstruction and in experimental models of breast cancer.

## ASCs and Adipocytes in Breast Cancer

### Normal Physiology and Obesity-Associated Changes

Adipocytes and ASCs represent significant components of breast tissue stroma with important endocrine signaling functions that contribute to both normal breast physiology and pathological processes such as the development of cancer (Wu et al., 2019). Breast tissue consists of many different specialized cell types, including contractile myoepithelial cells, luminal epithelial cells, and a well-organized stroma consisting of fibroblasts, adipocytes, and adipose stem cells. Complex interactions between epithelial cells and stromal tissue are crucial to establish and maintain normal tissue architecture during development and in physiologic circumstances where breast tissue undergoes remodeling, such as pregnancy and postpartum breast involution (Jindal et al., 2014; Soysal et al., 2015). Because adipocytes and ASCs are prevalent in the breast, we will compare the differential roles of each cell type in the normal breast and breast cancer setting.

Adipocytes constitute the major cellular component of adipose tissue and have important roles in both energy storage and endocrine functions. There are two major types of adipose tissues, each with different characteristics. Brown adipose tissue, characterized by abundant mitochondria, oxidizes lipids during cold-induced thermogenesis, whereas white adipose tissue, the primary type of adipose tissue in the breast, both insulates the body and acts as an energy reservoir through both storage of triglycerides and release of free fatty acids (Church et al., 2012; Hussain et al., 2020). In both tissue types, adipocytes respond to central nervous system signals by secreting peptide hormones and cytokines, collectively known as adipokines, to maintain energy balance and metabolic activity (Choe et al., 2016). Beyond metabolic regulation, the endocrine functions of adipocytes also include the production of estrone and estradiol in peripheral adipose tissues, which are integral to the well-regulated remodeling events that occur in the breast. In this way, adipocytes are essential to the development of the mammary gland during puberty and in the remodeling of breast tissue throughout the lactation cycle and involution (Colleluori et al., 2021).

In the context of obesity, the secretory profile of adipocytes is altered. In breast adipose tissue specifically, mRNA expression of aromatase correlated positively with BMI (Savolainen-Peltonen et al., 2018). Additionally, a survey of adipose depots in pre-menopausal women found that a higher waist circumference is associated with significantly increased estradiol in subcutaneous adipose tissue (Hetemäki et al., 2021). Increased aromatase and estrogen provide requisite ligands for estrogen receptor-positive breast cancer. Furthermore, hypertrophic obese adipocytes exhibit increased expression of pro-inflammatory cytokines, including TNF-alpha, IL-6, and IL-8 (Choe et al., 2016). These pro-inflammatory cytokines are associated with poor survival outcomes in breast cancer.

Adipose stem cells (ASCs) are subsets of mesenchymal stem cells with the multipotent capability to differentiate into many different cell types including osteocytes, chondrocytes, vascular endothelial cells, and adipocytes (Frese et al., 2016). Mesenchymal stem cells (MSCs), also known as mesenchymal stromal cells, are multipotent cells that have been implicated in the wound healing process and are commonly studied in regenerative medicine (Dai et al., 2016). While they were initially described as being present largely in the bone marrow, endogenous MSCs have been found in nearly all tissues (Bakker et al., 2010; Li et al., 2019). Furthermore, they have been found to interact with cells of the immune system during inflammation, proliferation, and tissue remodeling. Upon tissue injury, bone marrow MSCs facilitate the inflammatory process through secretion of cytokines, such as CCL-2, which causes extravasation of monocytes out of the bone marrow and into circulation, whereas activated resident tissue MSCs secrete inflammatory cytokines which attract neutrophils (Brandau et al., 2010; Li et al., 2019). After tissue damage, bone marrow MSCs (BM-MSCs) can also migrate from the bone marrow to a damaged region and differentiate into needed tissues and promote healing through paracrine signaling (Fu et al., 2019). Both exogenous and endogenous MSCs promote proliferation within damaged tissue through secretion of trophic factors (Li et al., 2019). ASCs are the primary type of MSC associated with breast tissue; they share stem cell surface markers, such as CD90, CD105, CD73, CD44, and CD166, with negative expressions of hematopoietic markers CD45 and CD34 (Bourin et al., 2013). Their ability to modulate the immune response has been associated with their regenerative role in damaged tissues (Frese et al., 2016). ASCs and MSCs have been studied extensively in the field of regenerative medicine: BM-MSCs are more popular in research, though the nature of their extraction from the marrow makes them relatively much more difficult to harvest and utilize than ASCs (Frese et al., 2016). ASCs, which are able to be harvested from subcutaneous fat, share not only the differentiation capacity of MSCs, but also their role in wound healing.

### Breast Cancer and the Obesity-Cancer Axis

The breast cancer tumor microenvironment is a complex system and shares many components with healthy breast tissue. It is composed of several specialized cell types, including adipocytes, ASCs, immune cells, cancer-associated fibroblasts, neoplastic cells, and the extracellular matrix (ECM) scaffold and stroma. Complex signaling events and interactions between different cell types in the tumor microenvironment contribute to the hallmarks of cancer, providing a supportive environment during the transformation of normal tissues into high-grade malignancies (Hanahan and Weinberg, 2011).

**Figure 1:**
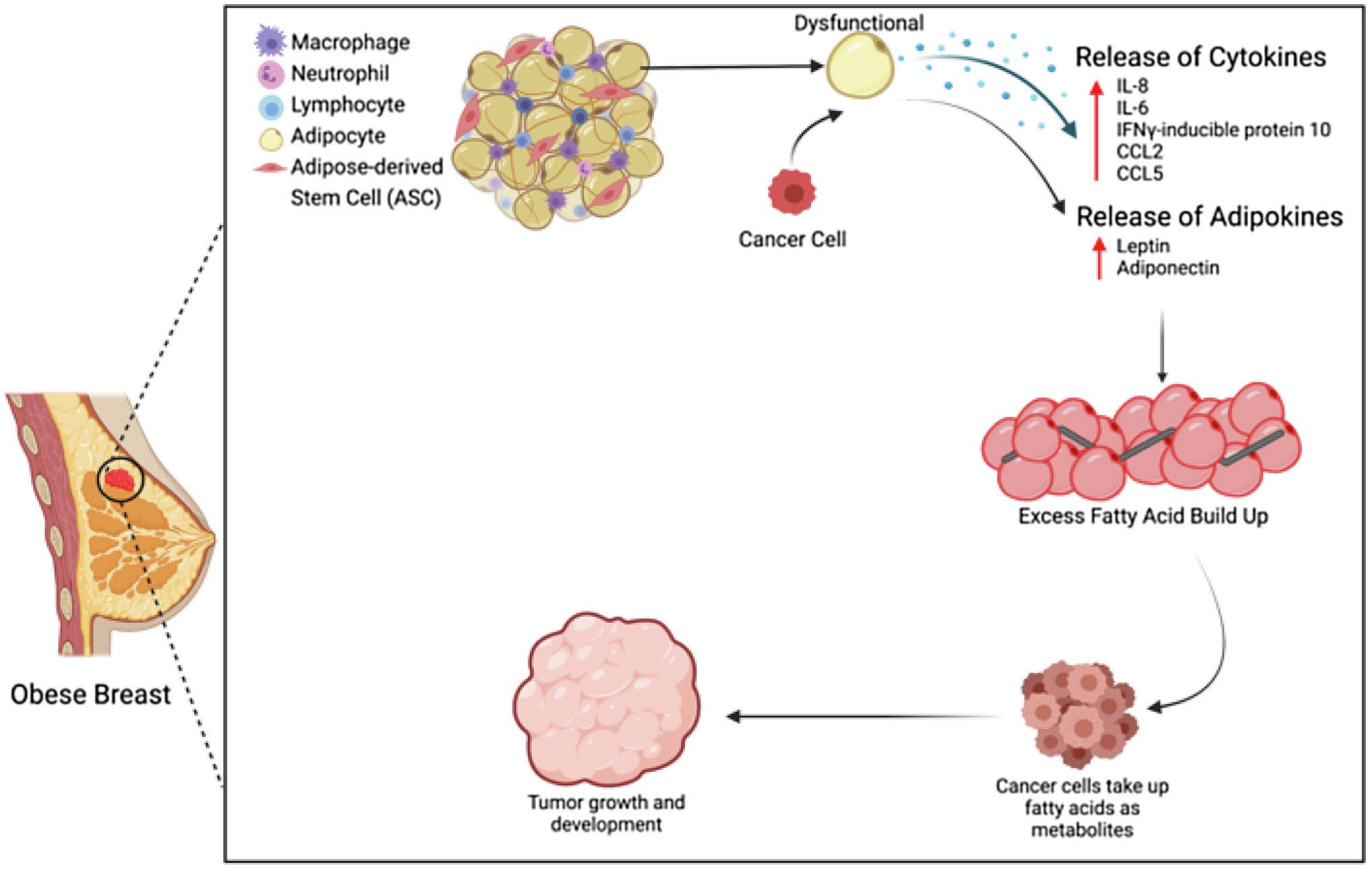
Adipocytes and ASCs in the obese tumor microenvironment. Adipocytes and ASCs contribute to the obese tumor microenvironment through increased secretion of cytokines and adipokines, as well as through increased fatty acid buildup.

Breast cancer grows in close proximity to adipose tissue, and cancer-associated adipocytes (CAAs) have been found to be a key player in breast cancer progression (Wu et al., 2019; Trivanović et al., 2020). Through their production of adipokines, adipocytes have the ability to regulate breast cancer through multiple mechanisms (Figure 1; Fujisaki et al., 2015). In samples of human breast cancer tissue, adipocytes located proximally to cancer cells were found to express IL-6 and mmp-11 *via* immunohistochemistry, whereas more distally located adipocytes did not (Dirat et al., 2011). Cytokines play a vital role in pro-inflammation, anti-infection, and other cellular signaling. When adipocytes are co-cultured with breast cancer cells, cytokines are secreted, specifically IL-8, IL-6, IFNγ-inducible protein 10, CCL2, and CCL5. These cytokines, when produced by immature adipocytes, can lead to an increased tumor initiation and metastasis in breast cancer (Picon-Ruiz et al., 2016). IL-6, IL-8, CCL2, and CCL5 have been found to be stimulators of the proliferation, survival, and invasion of breast cancer cells and are linked to an advanced stage of breast cancer (Nicolini et al., 2006; Soria and Ben-Baruch, 2008; Waugh and Wilson, 2008). Beyond the production of adipokines, adipocytes also have the ability to remodel the tumor microenvironment by releasing extracellular matrix proteins and recruiting other neighboring cells, such as macrophages and other immune cells, mimicking the tumor infiltrates (Chu et al., 2019).

The tumorigenic effects of adipocytes are further exacerbated by obesity. When adipocytes are dysfunctional, metabolic substrates, adipokines, and cytokines are released, leading to the promotion of proliferation, progression, invasion, and migration of breast cancer cells (Chu et al., 2019). Adipocytes play an important role in maintaining energy balance, so any adipocyte dysfunction will lead to excessive fatty acids, which can then be up-taken into the breast cancer tumors and thus serve as metabolites for tumor development through β-oxidation (Nieman et al., 2013). These fatty acids are the major source of ATP in the tumor (Choe et al., 2016). In addition to an increase of fatty acids, dysfunctional adipose tissue also increases cholesterol, triglycerides, hormones, and adipokines, which are all linked to metabolic dysfunction, insulin resistance, and resistance to cancer treatment. Insulin resistance can provide nutrients for cancer cell invasion, and when adipocytes are in co-culture with fibroblasts, any anti-cancer drug effects can be reduced through adipocytes metabolizing the drug into a less effective secondary product (Chu et al., 2019).

ASCs are a key component in the breast cancer tumor microenvironment and, through their interactions with the immune system and secretion of trophic factors, play integral roles in tumorigenesis, tumor growth, and distal metastasis. ASCs exhibit an immunomodulatory capacity and promote wound healing and regeneration in inflammatory environments (Sabol et al., 2018). However, this may have implications for cancer. One model depicts cancer as a wound that fails to heal while co-opting the wound healing response to promote their own growth (Dvorak, 2015). Interactions with the immune system can have a large effect on tumor development and growth: for example, CD4+CD25+FoxP3+ regulatory T cells that normally protect the host from autoimmune disease can get hijacked by cancers to suppress immune activity (Allen and Jones, 2010). ASCs are implicated in a variety of solid tumors, wherein their abnormal secretion profile has been shown to include tumorigenic factors. This trend continues in the context of breast cancer. ASCs have been found to secrete cytokines and growth factors, including leptin, IL-6, CXCL5, and PDGF (Sabol et al., 2019a). In a murine model of breast cancer, ASCs enhance breast cancer proliferation through secretion of IGF-1 (Fajka-Boja et al., 2020).

ASCs secrete a variety of interleukins, including IL-6, IL-7, IL-8, IL-11, and IL-12 (Zhao et al., 2018). IL-6 is involved in regulation of activation, growth, differentiation of immune cells during inflammatory, and immune responses. Furthermore, IL-6 has been reported to be strongly expressed by ASCs and is a major player in the proliferation, migration, and invasion of some cancer cells. In this context, ASCs have been shown to enhance the migration of ovarian cancer cells through an IL-6-mediated pathway. IL-6 secreted by ASCs activates the JAK2/STAT3 pathway and increases the level of phosphorylated JAK2 and STAT3, but it does not increase the total levels. The IL-6/JAK2/STAT3 pathway has been shown to increase proliferation and migration of ovarian cancer cells, and ovarian cancer patient derived ascites cells (Kim et al., 2017), as well as the migration and invasion of breast cancer cells (Walter et al., 2009).

When ASCs were co-cultured with breast cancer cell lines, there was a robust increase in cancer cell proliferation that was CXCL5-dependent (Zhao et al., 2018). CXCL5 is a chemokine previously linked to the chemotaxis of inflammatory cells. The secretory activity of ASCs considerably increases under hypoxia or tumorigenesis. Additionally, the interaction between tumor cells and adipose tissue can enhance CXCL5 expression. In *in vitro* studies with breast cells from tumor-free donors, ASCs significantly increased tumor cell numbers when co-cultured with breast cancer cell lines, independently of the presence of estrogen receptors. Analogously, ASC conditioned media has been reported to increase proliferation of breast cancer cells, but at a slower rate than cancer cells co-cultured with ASCs. Moreover, blocking CXCL5 with an anti-CXCL5 antibody was reported to greatly reduce the ability of ASCs to promote proliferation (Zhao et al., 2018).

Programmed cell death protein 4 (PDCD4) is a tumor suppressor gene, typically upregulated during apoptosis. Overexpression of PDCD4 in carcinoid cells has been linked to reduced proliferation of cancer cells (Göke et al., 2004). ASCs exposed to tumors can develop cytogenetic abnormalities and mesenchymal-to-epithelial transition (MET) (Elmageed et al., 2014). Exosomes from tumor cells have also been linked to genomic instability that leads ASCs to undergo oncogenic neoplastic transformations. These changes are associated with downregulation of the tumor suppressors, large tumor suppressor Homolog2 (Lats2), and PDCD4 (Elmageed et al., 2014). Such is the case of ASCs from patients with prostate cancer and of ASCs exposed to prostate cancer cells conditioned media. These altered ASCs were reported to express epithelial markers, such as androgen receptor (AR), prostate-specific antigen (PSA), CK8, and CK5/18, neoplastic features, including tumorigenic markers, such as Ki67, p53, and Ras, and vasculogenic markers, such as Willebrand factor and α-smooth muscle actin, alongside molecular features similar to those of prostate cancer tumor xenografts (Elmageed et al., 2014).

The capacity of ASCs to secrete pro-tumorigenic factors is increased in the context of obesity. ASCs harvested from obese individuals demonstrate increased expression of leptin, IL-1, IL-6, IL-12, PDGF-A, TNF-alpha, leukemia inhibitory factor, intercellular adhesion molecule 1, and granulocyte-colony stimulating factor compared to those harvested from lean individuals (Sabol et al., 2019a). Given the direct effects of obesity and adipose tissue on ASCs that drive aggressive breast cancer phenotypes, it is crucial to perform further studies to identify specific genes and proteins responsible for this phenotypic change. A key protein of interest in this field is leptin, a peptide hormone that is overexpressed in obesity.

Leptin, an adipokine expressed proportionally to fat mass, has a profound effect on prognosis (Abella et al., 2017). Increased leptin and leptin receptor expression is associated with worse prognosis, increased morbidity, mortality, metastasis, and cancer recurrence. This effect is present in many different cancer types, including bladder, lung, breast, prostate, testicular, ovarian, large B-cell lymphoma, mesothelioma, pancreatic, kidney, colorectal, liver, AML, and thyroid cancer (Lin et al., 2018). ASCs harvested from obese individuals (obASCs) generate increased leptin which leads to increased downstream estrogen levels and thus increased proliferation in the case of estrogen receptor-positive (ER+) breast cancer, with the non-estrogen leptin pathway promoting metastasis even in non-ER+ breast cancer (Sabol et al., 2019b). obASCs promote metastasis in triple-negative breast cancer cell lines by upregulating EMT genes and promoting migration *in vitro* (Sabol et al., 2019b). The pro-metastatic effects of obASCs were dependent upon leptin expression: Leptin KO *via* shRNA in obASCs abrogated metastatic effects both *in vitro* and *in vivo*. Leptin enhances EMT in TNBC through upregulation of Serpine1, Twist1, Snai2, IL-6, PTGS2, CCL5, and CD90 (Sabol et al., 2019b).

Leptin signaling has been shown to have immunomodulatory effects in the context of obesity: Malnutrition is associated with hypoleptinemia and increased susceptibility to infection, whereas obesity leads to hyperleptinemia, which is associated with an increase in autoimmune disorders and inflammation (Francisco et al., 2018). Long-term hyperleptinemia, observed in obesity, is associated with decreased natural killer cell immune activity, possibly due to leptin resistance (Li et al., 2016). Natural killer cells play a vital role in surveilling tissue to eliminate neoplastic growth. In the context of obesity, increased leptin signaling between ASCs and breast cancer cells represents an area where the proliferative and immunomodulatory effects of ASCs contribute to an environment that permits neoplastic growth.

Leptin is both an anorexigenic and pro-inflammatory factor and exists as a link between the neuroendocrine and immune systems. Leptin plays a role in both innate and adaptive immunity: It polarizes T helper cells toward a pro-inflammatory Th1 phenotype, which secretes IFN-γ, rather than the anti-inflammatory Th2 phenotype, which secretes IL-4 (Abella et al., 2017). Leptin has also been found to increase the expression of MMPs, specifically MMP-1, 3, 9, and 13, through the NF-κB, protein kinase C, and MAPK pathways (Abella et al., 2017). Beyond cancer, leptin may play a role in other diseases associated with inflammation: In non-alcoholic fatty liver disease, leptin activates hepatic stellate cells (Ito cells), leading to upregulation of pro-inflammatory and pro-angiogenic effects, acting as an inducer of hepatic fibrogenesis (Francisco et al., 2018). Obese patients with osteoarthritis have more leptin in their synovial fluid compared with lean patients. However, in murine models of osteoarthritis, obese leptin receptor-deficient mice, there was no difference in osteoarthritis rates compared to lean controls, indicating that leptin overexpression, and not necessarily obesity itself, is necessary for osteoarthritis development (Abella et al., 2017).

In the context of the immune system and breast cancer, leptin is hypothesized to induce IL-18 expression both in TAMs (tumor-associated macrophages) and breast cancer cells. Leptin-induced IL-18 expression was regulated *via* NF-κB/NF-κB1 signaling in TAMs and *via* PI3K-AKT/ATF-2 signaling in breast cancer cells, which eventually leads to invasion and metastasis in the latter. Effects of leptin on induction of NF-κB phosphorylation in TAMs were also significantly attenuated by a pharmacological NF-κB inhibitor, and for breast cancer cells MCF-7, PI3K inhibitor affected the decrease of Akt phosphorylation (Li et al., 2016). Increased IL-18 levels in the serum of cancer patients correlate with malignancy, and IL-18 acts as a crucial factor for cell migration in gastric cancer and melanoma. Through its impact on the immune system, secretion of leptin by ASCs impacts not only neoplastic cells, but also the resident tumor microenvironment.

There is also evidence that obesity contributes to activation of cancer stem cell signaling in breast cancer. Hillers-Ziemer et al. (2020) found that tumors grown within obese mice had increased expression of cancer stem cell-associated genes, such as SOX2 and NOTCH2, compared to those in lean mice. Additionally, Bowers et al. (2018) found that leptin signaling, which is increased in obASCs, contributes to obesity-mediated CSC enrichment. Tumors from obese mice had higher expression of cancer stem cell-associated genes, such as FOXC2 and TWIST2, while higher levels of the leptin expression found in the surrounding obese mammary fat pads. Furthermore, Bowers et al. found that breast cancer cell lines transfected with an shRNA-based knockdown of the leptin receptor had significant reduction in expression of cancer stem cell markers. As cancer stem cells play a critical role in chemoresistance and metastasis, these data may represent a mechanism by which obese-derived adipose stem cells mediate the cellular events responsible for increased mortality in obese breast cancer patients. However, additional research needs to be done to further establish the relationship between obASCs’ effect on cancer stem cells and increased metastasis in obesity.

Another instance in which obesity contributes to a tumor microenvironment that favors metastasis is through the increased capacity of ASCs from obese patients to differentiate into cancer/carcinoma-associated fibroblasts, or CAFs. CAFs display myofibroblast-like traits; they have been found in higher numbers in invasive tumors and enhance cancer cell proliferation, invasion, and metastasis (Hanahan and Weinberg, 2011; Karagiannis et al., 2012). They can originate from many diverse cell populations, including resident normal fibroblasts and ASCs; these cell types are transformed into CAFs when exposed to tumor derived factors (Strong et al., 2017; Liu et al., 2019). Obesity exacerbates this trend: Following exposure to breast cancer cells, obese ASCs expressed higher levels of CAF markers, including alpha-SMA, NG2, FAP, and FSP, compared to ASCs from lean individuals (Strong et al., 2017). This is another example of how cross talk between breast cancer cells and stromal cells creates a pro-tumor environment: Cancer cells transform stromal cells into CAFs, and CAFs secrete unique cytokines and growth factors, such as CXCL12, VEGF, PDGF, and hepatocyte growth factor (Plaks et al., 2015; De Palma et al., 2017). Co-culture with obese ASCs resulted in breast cancer cells having increased mesenchymal phenotype and proliferative capacity compared to those cultured with lean ASCs, indicating an increased capacity for tumor growth and metastasis. Due to CAFs’ role in supporting tumor growth, it is critical to identify factors that contribute to the development of this population in the tumor microenvironment. Furthermore, there is substantial evidence demonstrating that CAFs are important regulators of the cancer immune response and promote immune evasion of cancer cells (Liu et al., 2019).

Obesity further promotes breast cancer tumorigenic potential by remodeling the extracellular matrix (ECM). Tumor-associated ASCs increase the expression and stiffness of fibronectin *via* mediation of secreted TGF-B in triple-negative breast cancer (Chandler et al., 2011). Seo et al. found that ASCs isolated from adipose tissue in obese versus lean mice create a stiffer matrix by promoting increased myofibroblast differentiation through mechanotransduction (Seo et al., 2015). This altered matrix contains increased concentrations of aligned collagen-1 and fibronectin fibers and can promote the malignant potential of mammary epithelial cells (Springer et al., 2019). Interstitial fibrosis associated with obesity also contributes to a phenotypic change in macrophages that resembles that of TAMs (Dunne et al., 2014). Bone marrow-derived macrophages cultured on obese versus lean ASC matrix have increased expression of anti-inflammatory macrophage (M2) markers CD206 and arginase-1 (Dunne et al., 2014). Furthermore, transcriptomic data indicate that macrophages from obese versus lean tumor-free human breast adipose have increased expression of genes that are associated with the TAM phenotype (Dunne et al., 2014). These data collectively suggest that obesity-associated ECM can promote tumorigenic potential by recapitulating characteristics of tumor-associated stroma.

## ASCs in Novel Laboratory Models of Breast Cancer

### 3D Model Systems

**Figure 2:**
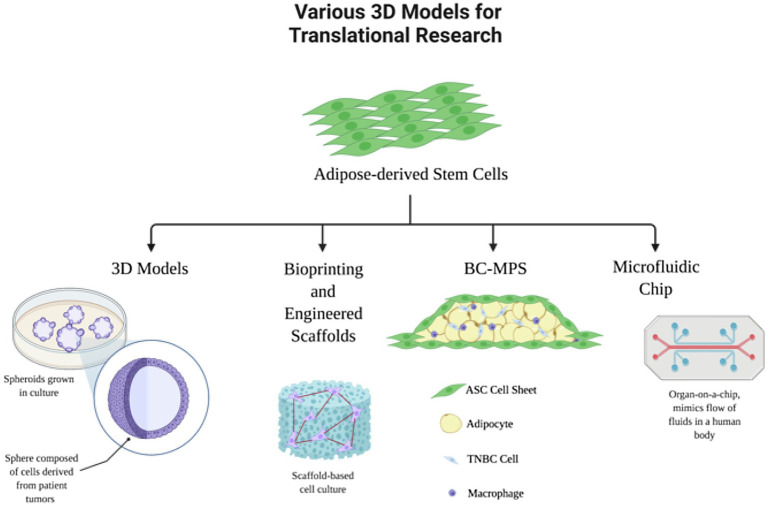
Adipose-derived stem cells in translational research models. Adipocytes and ASCs have many applications in 3D laboratory models of breast cancer.

In cancer research, *in vitro* 2D cell cultures are most commonly used for understanding cellular biology and morphology, as well as in pre-clinical drug trials (Jacoby and Pasten, 1979). However, there are many disadvantages and limitations with 2D cultures, such as no cell-to-extracellular interactions and cell morphology changes (Kapałczyńska et al., 2018). These limitations of 2D culture systems are addressed by 3D culture systems, because they more closely mimic the behavior of the cells in their natural environment: the human body. 3D cultures can be advantageous over 2D cultures in that these environmental niches are replicated. Figure 2 summarizes the applications of ASCs in 3D laboratory models of breast cancer. For example, spheroids are 3D spheres made up of multiple layers of cells, a model which mimics the characteristics of a solid tumor mass (Kapałczyńska et al., 2018). This spheroid structure introduces cell-to-cell and cell-to-environment interactions in actual tissues relative to 2D cultures.

3D cancer models can provide more accurate results for drug responses and identification of biomarkers (Ravi et al., 2017). In addition, since spheroids allow local stroma and ECM interactions, these models have been used to show what drives malignant tumor cells to proliferate, invade, and migrate. Research has shown that tumors commonly grow near adipose tissue, and thus, there is a direct relationship to growth of cancer cells. Adipocyte dysfunction has even more directly been related to breast cancer, regulating cancer cells and increasing tumor progression (Choi et al., 2018). As a result, 3D model systems are favorable in order to study the cancer-to-adipocyte interaction. Human ASCs are commonly used in these 3D spheroids to develop an adipocyte microtissue, creating a native adipose tissue-like ECM (Hoefner et al., 2020). These ASC spheroids have been used in combination with 3D bioprinted constructs to use for a 3D breast cancer-adipose tissue model. Horder et al. demonstrated remodeling of the adipose tissue ECM and tumor cell-induced modulation of the lipid content when co-cultured with breast cancer cells by using a 3D printed tissue construct with MDA-MB-231 cells in combination with ASC spheroids. Results showed an increase in fibronectin, collagen I, and collagen VI expression, demonstrating that 3D model systems more accurately represent the complexity of the cellular and matric cross talk within the tumor-stroma microenvironment (Horder et al., 2021). More recently, these 3D spheroids are used in combination with engineered 3D printed scaffolds using biodegradable materials.

### 3D Bioprinting and Engineered Scaffolds

Biotechnology and fabrication have recently emerged as a superior approach to creating microphysiological systems in order to study different diseases in the human body, especially cancer. Because human tissues are organized in a complex 3D structure with cellular-ECM cross talk, 3D bioprinting allows for the design of a complex architecture with placement of the cellular components in physiological spatial micro-arrangements (Horder et al., 2021). Many different approaches can be taken to create 3D bioprinted cellular models: Individual cells can be printed in hydrogel-based inks, multicellular aggregates, such as spheroids, and complex engineered 3D constructs that mimic microtissues can be bioprinted (Benmeridja et al., 2020; Levato et al., 2020).

Tissue-engineered constructs, which utilizes a biocompatible or biodegradable material to engineer a scaffold, is another novel technique used to recreate the breast mound post-mastectomy. In this technique, the ASCs are seeded on scaffold-based tissue-engineered constructs in which the ASCs will differentiate into adipocytes and form ECM, creating sheets which can be formed into thicker adipose constructs. The design and scaffold material used is important in order to overcome issues related to volume retention and vascularization (Zhang et al., 2009). Scaffolds allow for cells to be cultured in a 3D microenvironment in order to more accurately mimic the native tissue *in vivo*. There are many factors to consider when designing the scaffold: scaffold pore size (highly porous structure is required for vascular ingrowth and cell differentiation), stiffness, flexibility, biomaterials used, biomechanical properties, and degradation properties of the biomaterial (Stumpf et al., 2020). The advantage of the scaffolds is the biocompatibility and ability to interact with ASCs. One main challenge with these scaffolds is the vascularization of the regenerated tissue. In addition, this method requires long-term maintenance of larger tissue volumes (Petit et al., 2012).

Decellularized extracellular matrix (ECM) is another commonly used technique when designing tissue-engineered scaffolds. These decellularized ECM scaffolds when re-cellularized with the recipient patient’s ASCs (commonly used in cellular therapy due to their capability to differentiate into MSCs and ESCs) can be advantageous for personalized medicine in autologous applications (Gentile et al., 2020). The ECM is also a major component of the tumor microenvironment because it promotes local invasion and drives metastasis. Wishart et al. show that decellularized ECM from tumor-bearing and obese mammary glands drive triple-negative breast cancer (TNBC) cell invasion. By using proteomics, this decellularized ECM scaffold enabled Wishart to identify collagen VI as a driving force behind TNBC invasion *via* cross talk between NG2-EGFR and MAPK signaling (Wishart et al., 2020). Engineering decellularized ECM scaffolds from different tissues is a valuable method for identifying ECM functions. Dunne et al. show that decellularized human adipose tissue can be used as an *in vitro* model to recapitulate luminal and HER2+ breast cancer cell growth and drug sensitivity that is seen *in vivo*. MCF-7, BT474, and SKBR3 cells cultured on decellularized adipose scaffolds exhibited different proliferation, migration, and morphological profiles than cells cultured in 2D conditions (Dunne et al., 2014). Furthermore, these cell lines exhibited either reduced or elevated drug sensitivity to doxorubicin (chemotherapeutic) or lapatinib (an EGFR inhibitor) when on a decellularized adipose scaffold than in 2D culture (Dunne et al., 2014). These data indicate a microenvironment-dependent function of these breast cancer cell lines.

### White Adipose Tissue Microphysiological Systems

White adipose tissue (WAT) inflammation has been found to have a positive relationship with breast cancer. Thus, WAT has become increasingly common for use when researching novel targets in breast cancer. Zhao et.al investigated the relationship between breast WAT inflammation, obesity, and breast cancer. Previous studies show that WAT inflammation, defined by the presence of CLS-B, is formed by dying or dead adipocytes surrounded by macrophages. It was found that WAT inflammation usually occurs in overweight and obese breast cancer patients (Iyengar et al., 2016; Zhao et al., 2020). WAT has been used in 3D model systems to study different diseases, such as type 2 diabetes: Membrane mature adipocyte aggregate cultures (MAAC) cultures freshly isolated adipocytes under a permeable small-pored membrane insert in order to facilitate access to nutrients and oxygen (Harms et al., 2019). This method allows for long-term culture of the adipocytes to study long-term effects on WAT. In another 3D microphysiological system, primary WAT is sandwiched between tissue-engineered sheets of adipose-derived stromal cells (ASCs) creating “sandwiched white adipose tissue” (SWAT) which can be used to study long-term studies on WAT physiology and pathology as well as personalized medicines and drug development (Lau et al., 2018). In more recent developments, this system has been used to create a microphysiological system to study breast cancer (BC-MPS) by seeding breast cancer cells on the primary white adipose tissue. This system was found to capture the cross talk between healthy primary human breast mammary breast adipose tissue and breast cancer cells (Brown et al., 2021). WAT has also been used in 3D microfluidic models or organ-on-a-chip devices to study different diseases, such as diabetes or cachexia (Kongsuphol et al., 2019; Rogal et al., 2020).

### Microfluidic Chip Systems

Organ-on-a-chip models have recently become a promising alternative to non-human animal models. By adding microfluidics into microphysiological systems, a more physiologically relevant human microenvironment is created because the human tissue is supplied by a vasculature-like microfluidic perfusion (Rogal et al., 2020). For example, Rogal et al. outlines a novel WAT-on-a-chip multilayer device which incorporated chambers specifically for white adipose tissue with different channels for media flow in order to provide nutrients for the WAT. Since WAT is a key contributor to many metabolic diseases, this device provides a model for studying multiple diseases as well as pharmaceutical discovery. These chip systems are an especially effective development for human oncology since the addition of microfluidics mimic the flow of tumor microenvironment at the micrometer scale and are capable of controlling physicochemical properties, such as interstitial pressure, soluble gradient factors, and oxygen tension (Del Piccolo et al., 2021). The tumor microenvironment is incredibly complex, and tumor cells can spread from the primary affected organ to other parts of the body through multiple steps known as the metastatic cascade. A critical step of the metastatic cascade involves tumor cells going through an epithelial-mesenchymal transition (EMT) to become invasive and migratory. The microfluidic organ-on-a-chip systems are beneficial when studying EMT and migration, as they mimic the physicochemical properties of the tumor microenvironment. Ultimately, these 3D microphysiological systems (spheroids, scaffolds, and organ-on-a-chip systems) all provide a system that more closely mimics the human body by using engineered human tissues and creating a physiological microenvironment, and thus are advantageous to breast cancer therapy.

## ASC Suitability for Tissue Engineering

### Oncological Safety

Adipose-derived stem cells have emerged as key players in developing novel tissue engineering techniques, even more so in the field of breast reconstruction. However, a main concern is the oncological safety of ASC therapies or surgical implantation of the engineered constructs. ASCs have the potential to influence the behavior of breast cancer cells due to secreted adipokines and their effect on the tumor microenvironment (O’Halloran et al., 2017). Furthermore, as mentioned in the above sections, adipokines, which are involved in the promotion of tumor growth, are pro-inflammatory and secreted in an increased amount in obese individuals, which may place obese individuals at a greater risk of cancer recurrence when using ASCs in breast reconstruction. However, while it is clear that ASCs have a role in breast cancer progression, there is no evidence that ASCs have an effect on the growth and progression of distant tumors when used in wound repair (Yarak and Okamoto, 2010). It has been demonstrated in murine models that dermal wound microenvironments can retain ASCs, preventing them from migrating to distant neoplastic foci and causing tumor progression (Altman et al., 2011). This study suggests adipose-derived stem cell therapy for reconstructive surgery is a feasible and safe method in a malignant environment. While this is promising, it is clear that more research is needed to fully resolve questions of safety.

### Novel Approaches to Breast Reconstruction: Autologous Fat Grafting

ASC-enhanced fat grafting is a novel alternative to prosthetic implants for breast reconstruction for patients who have undergone a mastectomy. This method utilizes tissue engineering strategies by using ASCs to generate adipose tissue in the breast to reaugment the breast post-mastectomy (O’Halloran et al., 2017; Herly et al., 2018). However, the efficacy of this method is questionable as one study reported an increased rate of local recurrence of up to 10.8%, although this study specifically sampled patients with intraepithelial neoplasia and there are limited published studies that broadly account for breast cancer recurrence statistics after autologous fat grafting (O’Halloran et al., 2017; Herly et al., 2018). However, Stumpf et al. reported after a 5-year follow-up on a total of 320 patients, there was no significant difference in locoregional recurrence rates found between patients who had received immediate AFG (autologous fat grafting) and those who underwent breast-conserving surgery alone (Petit et al., 2012, 2013). This study further supports that immediate AFG reconstruction is an oncologically safe technique for women who have undergone a mastectomy due to breast cancer. There are many factors that still need to be considered for AFG reconstruction in order to prevent localized recurrence: the timing of AFG after surgery, adequate follow-up, use of low-risk vs. high-risk patients, etc. (Wise et al., 2015). However, the biggest challenge with autologous fat grafting is being able to utilize large concentrations of ASCs in order to generate a large amount of adipose tissue (Zhang et al., 2009).

## Conclusion and Future Directions

In this review, we have delineated adipocytes’ and ASCs’ many physiological and pathological roles, including breast cancer. They are a key component of the breast tumor microenvironment and provide both proliferative cues and drive carcinogenesis, especially in the context of obesity. Future directions into this area of research include using pharmacological or molecular inhibitors of downstream targets of ASCs to test their direct effects on cancer development and progression. We have described adipocytes and ASCs in novel laboratory models of breast cancer. Novel microphysiological systems enable more translational research of complicated cell-to-cell and cell-to-tissue interactions that occur in the human body. These 3D models can range from spheroids, 3D printed scaffolds or constructs, microphysiological systems to organ-on-a-chip systems, which are useful for studying the mechanisms behind cancer invasion and migration with implications for pharmaceutical discovery and targeted cancer therapy. Adipocytes and ASCs are also promising tools in cancer therapeutics and regenerative medicine, due to their plastic nature. Further investigation is required to ensure the safety of ASC use in tissue-engineered constructs and/or autologous grafting.

## Author Contributions

CB and KH wrote this manuscript with help of MA, MW, and TC. KH and GW created figures. KN and MSA contributed to the final revision of this manuscript. BC-B, BB, and MB contributed financially to the manuscript. All authors contributed to the article and approved the submitted version.

## Funding

This project received funding from the National Institutes of Health 1R01CA174785-01A1 (BC-B) and 1R41CA257425-01 (MB). The project was also supported by Award Number TL1TR003106 from the National Center for Advancing Translational Sciences. The content is solely the responsibility of the authors and does not necessarily represent the official views of the National Center for Advancing Translational Sciences or the National Institutes of Health.

## Conflict of Interest

The authors declare that the research was conducted in the absence of any commercial or financial relationships that could be construed as a potential conflict of interest.

## Publisher’s Note

All claims expressed in this article are solely those of the authors and do not necessarily represent those of their affiliated organizations, or those of the publisher, the editors and the reviewers. Any product that may be evaluated in this article, or claim that may be made by its manufacturer, is not guaranteed or endorsed by the publisher.
